# Clinical relevance of genetic polymorphisms in WNT signaling pathway (*SFRP1*, *WNT3A*, *CTNNB1, WIF-1*, *DKK-1*, *LRP5*, *LRP6*) on pulmonary tuberculosis in a Chinese population

**DOI:** 10.3389/fimmu.2022.1011700

**Published:** 2022-12-07

**Authors:** Qian Huang, Chao-Cai Wang, Yun-Guang Liu, Chang-Ming Zhao, Tian-Ping Zhang, Yan Liu, Hua Wang

**Affiliations:** ^1^ Department of Public Health, Medical College of Qinghai University, Xining, China; ^2^ Department of Infection Disease, Qinghai Center for Disease Prevention and Control, Xining, China; ^3^ The First Affiliated Hospital of USTC, Division of Life Sciences and Medicine, University of Science and Technology of China, Hefei, Anhui, China; ^4^ Department of Tuberculosis, Anhui Chest Hospital, Hefei, Anhui, China

**Keywords:** pulmonary tuberculosis, infectious disease, *Mycobacterium tuberculosis*, WNT signaling pathway, single nucleotide polymorphisms

## Abstract

The present study was performed to evaluate the association of WNT signaling pathway genes variants with pulmonary tuberculosis (PTB) risk in Chinese Han population. Our study subjects were composed of 452 PTB patients and 465 normal controls, and seventeen SNPs of seven genes in WNT signaling pathway (*SFRP1*, *WNT3A*, *CTNNB1, WIF-1*, *DKK-1*, *LRP5*, *LRP6*) were genotyped by SNPscan technique. We found no significant relationship of *SFRP1* rs10088390, rs4736958, rs3242, *WNT3A* rs752107, rs3121310, *CTNNB1* rs2293303, rs1798802, rs4135385, *WIF-1* rs1026024, rs3782499, *DKK-1* rs2241529, rs1569198, *LRP5* rs3736228, rs556442, *LRP6* rs2302685, rs11054697, rs10743980 polymorphisms with PTB susceptibility. While, *WIF-1* rs3782499 variant was associated with susceptibility to PTB under recessive model, and haplotype analysis showed that *DKK-1* GA haplotype frequency was significantly increased in PTB patients. The *WNT3A* rs3121310, *CTNNB1* rs2293303 polymorphisms were respectively associated with drug-induced liver injury (DILI), sputum smear-positive in PTB patients. The rs3782499 in *WIF-1* gene was related to fever, leukopenia, and the rs1569198 in *DKK-1* was linked to sputum smear-positive in PTB patients. In *LRP5* gene, rs3736228, rs556442 variants respectively affected the occurrence of DILI, fever, and *LRP6* gene rs2302685, rs10743980 variants respectively influenced the development of hypoproteinemia, sputum smear-positive in PTB patients. Our results revealed that WNT signaling pathway genes variation were not associated with the susceptibility to PTB, while *WNT3A*, *CTNNB1*, *WIF-1*, *DKK-1*, *LRP5*, *LRP6* genetic variations might be closely related to the occurrence of several clinical characteristics of PTB patients.

## Introduction

Tuberculosis (TB) remains a leading infectious cause of morbidity and mortality caused by infection of *Mycobacterium tuberculosis* (MTB) in developing countries, with an estimated 9.9 million new cases worldwide in 2021 (1). Epidemiological studies had shown that more than one-third of the global population were infected with MTB, while only 5-10% of infections eventually developed into active TB (2, 3). This suggested that the progression of TB disease was affected environment elements, pathogenic features of MTB, and the host genetic factors (4, 5). Previous association studies had assessed some candidate loci that might be associated with TB susceptibility, including the genes encoding human leukocyte antigen, cytokines and chemokines (6, 7). However, these studies only investigated a few polymorphisms, and explained only part of TB heritability. On the other hand, pathway-based association studies based on the biological functions of related signaling pathway networks were considered to be an effective method to identify the pathogenic genes of complex diseases. Several molecular pathways that regulated immune response, such as JAK/STAT, MAPK, NF-KB signaling, had been the hot area in the studies regarding TB infection and susceptibility (8, 9).

The WNT signaling pathway was considered as an important transduction cascade modulating host immune responses against microbial pathogens, governing ontogeny and homeostatic processes (3). The canonical WNT signaling pathway was activated when WNT proteins bind to LRP5/6-Frizzled receptors and co-receptors, which caused β-catenin co-activating transcription factors to affect the downstream gene expression (10). Recent evidences implicated that the WNT pathway played an etiological role in the pathogenesis of TB. Wu et al. found that the activation of Wnt/β-catenin signaling could induce mycobacteria-infected cell apoptosis and promote the secretion of pro-inflammatory tumor necrosis factor (TNF)-α, interleukin (IL)-6 (11). Another study by Schaale et al. revealed that the mRNA expression of most WNT homologous were significantly decreased during MTB infection, and further suggested that WNT6 was expressed in granulomatous lesions and had a key role in macrophage differentiation and proliferation (12). The specific molecular mechanism of WNT signaling pathway in the pathogenesis of PTB had attracted extensive attention, but it was still poorly understood and needed further exploration.

Genetic variations in WNT signaling pathway genes were proved to be associated with susceptibility to a variety of human diseases, such as infectious diseases and cancer, implying that polymorphisms in the WNT pathway might also affect susceptibility to PTB (3, 13, 14). Hu et al. previously found multiple single nucleotide polymorphisms (SNP) in WNT pathway genes were associated with PTB susceptibility in Western Chinese (3). It was important to note that additional studies were needed to validate these results, however, there were few studies on the epidemiological genetic association between WNT signaling pathway genes and PTB susceptibility. In order to explore the potential roles of WNT pathway genetic polymorphisms in PTB, this study was designed to evaluate the associations of some SNPs from seven key genes in WNT signaling pathway (*SFRP1, WNT3A, CTNNB1, WIF-1, DKK-1, LRP5, LRP6*) with PTB susceptibility in a Chinese population.

## Materials and methods

### Study participants

In the present study, the PTB patients were selected from the Department of Tuberculosis at Anhui Chest Hospital, and unrelated healthy individuals were recruited from the health examine center as control group subjects. All the subjects were in the same region, and the Han Chinese population. The diagnosis of PTB patients was based on the following criteria: suspicious clinical symptoms, chest radiography, sputum and/or bronchoalveolar lavage fluid MTB culture, microscopy for acid fast bacilli (AFB), and effect of anti-TB treatment. In addition, the exclusion criteria for PTB patients including HIV positive, hepatitis, malignancy, immune deficiency, and other infectious disease. All controls were required to have no history of TB, infectious diseases or malignant neoplasms, negative sputum smear and culture, and normal chest radiographs.

Our study was approved by the Ethics Committee of Anhui Chest Hospital, and followed the Declaration of Helsinki protocols. The written informed and consent was provided by every participant prior to enrollment in the study. Then, the peripheral blood samples, demographic characteristics, and clinical data of study participants were collected. The required clinical information for PTB patients included fever (≥ 37.3°C), drug resistance, drug-induced liver injury (DILI), pulmonary infection, hypoproteinemia (Serum total protein < 60 g/L or albumin < 35 g/L), leukopenia (peripheral blood white blood cell count < 4.0×10^9/^L), sputum smears-positive (the amount of bacteria ≥10^4^/mL), *etc.*

### SNP selection and genotyping

Seven genes in WNT signaling pathway (*SFRP1*, *WNT3A*, *CTNNB1*, *WIF-1*, *DKK-1*, *LRP5*, *LRP6*) were selected for analyses in the present study. The genetic variation information of these genes in Han Chinese people in Beijing was obtained by the Ensembl Genome Browser 85 and CHBS_1000g, and HaploView 4.0 software (Cambridge, MA, USA) was used to select the tag SNPs of each gene. Then, we searched the existing researches regarding the association between these genes and human diseases, and looked for the functional tag SNPs related to human disease. Finally, we selected three SNPs (rs10088390, rs4736958, rs3242) in *SFRP1* gene, two SNPs (rs752107, rs3121310) in *WNT3A* gene, three SNPs (rs2293303, rs1798802, rs4135385) in *CTNNB* gene, two SNPs (rs1026024, rs3782499) in *WIF-1* gene, two SNPs (rs2241529, rs1569198) in *DKK-1* gene, two SNPs (rs3736228, rs556442) in *LRP5* gene, three SNPs (rs2302685, rs11054697, rs10743980) in *LRP6* gene, respectively. Above SNPs satisfied these conditions: minor allele frequency (MAF) ≥ 0.05 in CHB and *r^2^* threshold > 0.8.

We collected the peripheral blood samples from each subject by tubes containing ethylenediaminetetraacetic acid with median cubital vein, and extracted the genomic DNA from peripheral blood by the Flexi Gene-DNA Kit (Qiagen, Valencia, CA). The SNPscan technique was applied for genotyping with the technical support of the Center for Genetic & Genomic Anal-ysis, Genesky Biotechnologies (Inc., Shanghai), and the specific experimental steps could be found in the previous study. Only the subject with 100% success in SNP genotyping were included in the final analysis.

### Statistical analysis

All the statistical analysis was performed by SPSS 23.0 (SPSS Inc, IL, USA) in this study. The Hardy-Weinberg equilibrium test of candidate SNPs was evaluated by Chi-square among normal controls. We analyzed the differences of genotype distribution, and allele frequency of all SNPs between the PTB group and control group by Chi-square test, and the associations between these SNPs and the clinical features of PTB patients were also analyzed using Chi-square test. Moreover, logistic regression model was used to calculate the odds ratios (OR) and 95% confidence intervals (CI). The relationship between each SNP and PTB risk in dominant and recessive model was also explored, and haplotype frequencies between PTB group and control group was also estimated by SHEsis (15). A *P* value < 0.05 was considered as the statistically significant.

## Results

### Association of WNT signaling pathway genes SNPs with PTB

This study included 452 PTB patients (192 females and 260 males) and 465 normal controls (262 females and 203 male), and the mean age of PTB patients and normal controls was 44.88 ± 17.32, 44.12 ± 13.85, respectively. The allele, genotype frequencies of all SNPs in *SFRP1*, *WNT3A*, *CTNNB1*, *WIF-1*, *DKK-1*, *LRP5*, *LRP6* genes are summarized in **Table 1**, and the genotype distribution of all SNPs in control met Hardy Weinberg equilibrium.

**Table 1:** Comparison of WNT pathway genes variations between PTB patients and controls.

SNP	Analyze model		PTB patients	Controls	*P* value	*OR* (95% *CI*)
*SFRP1*
rs10088390	Genotypes	GG	67 (14.82)	62 (13.33)	0.566	1.126 (0.751, 1.690)
		CG	219 (48.45)	230 (49.46)	0.957	0.992 (0.748, 1.316)
		CC	166 (36.73)	173 (37.20)	Reference
	Alleles	G	353 (39.05)	354 (38.06)	0.665	1.026 (0.914,1.152)
		C	551 (60.95)	576 (61.94)	Reference
	Dominant model	CC	166 (36.73)	173 (37.20)	0.881	0.987 (0.834, 1.169)
		CG+GG	286 (63.27)	292 (62.80)	Reference
	Recessive model	GG	67 (14.82)	62 (13.33)	0.517	1.112 (0.807, 1.531)
		CG+CC	385 (85.18)	403 (86.67)	Reference
rs4736958	Genotypes	CC	48 (10.62)	42 (9.03)	0.567	1.143 (0.724,1.804)
		TC	197 (43.58)	216 (46.45)	0.508	0.912 (0.694, 1.198)
		TT	207 (45.80)	207 (44.52)	Reference
	Alleles	C	293 (32.41)	300 (32.26)	0.944	1.005 (0.880, 1.147)
		T	611 (67.59)	630 (67.74)	Reference
	Dominant model	TT	207 (45.80)	207 (44.52)	0.697	0.977 (0.869, 1.099)
		CC+TC	245 (54.20)	258 (55.48)	Reference
	Recessive model	CC	48 (10.62)	42 (9.03)	0.419	1.176 (0.793, 1.742)
		TC+TT	404 (89.38)	423 (90.97)	Reference
rs3242	Genotypes	AA	3 (0.66)	1 (0.22)	0.334	3.059 (0.371, 29.529)
		AG	42 (9.29)	49 (10.54)	0.543	0.874 (0.566, 1.349)
		GG	407 (90.05)	415 (89.25)	Reference
	Alleles	A	48 (5.31)	51 (5.48)	0.869	1.002 (0.980, 1.024)
		G	856 (94.70)	879 (94.52)	Reference
	Dominant model	GG	407 (90.05)	415 (89.25)	0.692	0.9260.623, 1.356)
		AA+AG	45 (9.96)	50 (10.75)	Reference
	Recessive model	AA	3 (0.66)	1 (0.22)	0.303	3.086 (0.322, 29.560)
		AG+GG	449 (99.34)	464 (99.78)	Reference
*WNT3A*						
rs752107	Genotypes	TT	28 (6.19)	25 (5.38)	0.765	1.090 (0.619, 1.920
		TC	161 (35.62)	184 (39.57)	0.249	0.852 (0.648, 1.119)
		CC	263 (58.19)	256 (55.05)	Reference
	Alleles	T	217 (24.00)	234 (25.16)	0.565	1.015 (0.964, 1.070)
		C	687 (76.00)	696 (74.84)	Reference
	Dominant model	CC	263 (58.19)	256 (55.05)	0.339	0.930 (0.802, 1.079)
		TC+TT	189 (41.82)	209 (44.95)	Reference
	Recessive model	TT	28 (6.19)	25 (5.38)	0.595	1.152 (0.683,1.945)
		CC+TC	424 (93.81)	440 (94.62)	Reference
rs3121310	Genotypes	AA	73 (16.15)	66 (14.19)	0.518	1.139 (0.767, 1.692)
		AG	213 (47.12)	228 (49.03)	0.791	0.962 (0.725, 1.278)
		GG	166 (36.73)	171 (36.77)	Reference
	Alleles	A	359 (39.71)	360 (38.71)	0.660	1.026 (0.915, 1.150)
		G	545 (60.29)	570 (61.30)	Reference
	Dominant model	GG	166 (36.73)	171 (36.77)	0.988	0.999 (0.843, 1.184)
		AA+AG	286 (63.27)	294 (63.23)	Reference
	Recessive model	AA	73 (16.15)	66 (14.19)	0.409	1.138 (0.837, 1.546)
		AG+GG	379 (83.85)	399 (85.81)	Reference
*CTNNB1*
rs2293303	Genotypes	TT	12 (2.65)	7 (1.51)	0.200	1.855 (0.721, 4.767)
		CT	110 (24.34)	101 (21.72)	0.298	1.178 (0.865, 1.604)
		CC	330 (73.01)	357 (76.77)	Reference
	Alleles	T	134 (14.82)	115 (12.37)	0.125	1.199 (0.951, 1.511)
		C	770 (85.18)	815 (87.63)	Reference
	Dominant model	CC	330 (73.01)	357 (76.77)	0.188	0.951 (0.882, 1.025)
		CT+TT	122 (26.99)	108 (23.22)	Reference
	Recessive model	TT	12 (2.65)	7 (1.51)	0.222	1.764 (0.701, 4.439)
		CC+CT	440 (97.35)	458 (98.49)	Reference
rs1798802	Genotypes	AA	42 (9.29)	41 (8.82)	0.474	1.187 (0.742, 1.900)
		AG	209 (46.24)	191 (41.08)	0.087	1.268 (0.966, 1.665)
		GG	201 (44.47)	233 (50.11)	Reference
	Alleles	A	293 (32.41)	273 (29.35)	0.157	1.104 (0.963, 1.266)
		G	611 (67.59)	657 (70.65)	Reference
	Dominant model	GG	201 (44.47)	233 (50.11)	0.087	0.887 (0.774, 1.018)
		AA+AG	251 (55.53)	232 (49.89)	Reference
	Recessive model	AA	42 (9.29)	41 (8.81)	0.802	1.054 (0.699, 1.589)
		AG+GG	410 (90.71)	424 (91.18)	Reference
rs4135385	Genotypes	AA	104 (23.01)	96 (20.65)	0.196	1.282 (0.880, 1.869)
		AG	239 (52.88)	240 (51.61)	0.301	1.179 (0.863, 1.609)
		GG	109 (24.12)	129 (27.74)	Reference
	Alleles	A	447 (49.45)	432 (46.45)	0.199	1.064 (0.968, 1.171)
		G	457 (50.55)	498 (53.55)	Reference
	Dominant model	GG	109 (24.12)	129 (27.74)	0.210	0.869 (0.698, 1.083)
		AA+AG	343 (75.88)	336 (72.26)	Reference
	Recessive model	AA	104 (23.01)	96 (20.65)	0.386	1.114 (0.872, 1.424)
		AG+GG	348 (76.99)	369 (79.35)	Reference
*WIF-1*						
rs1026024	Genotypes	AA	14 (3.10)	12 (2.58)	0.789	1.130 (0.507, 2.447)
		AG	131 (28.98)	160 (34.40)	0.085	0.781 (0.590, 1.035)
		GG	307 (67.92)	293 (63.01)	Reference
	Alleles	A	159 (17.59)	184 (19.78)	0.228	1.027 (0.983, 1.073)
		G	745 (82.41)	746 (80.22)	Reference
	Dominant model	GG	307 (67.92)	293 (63.01)	0.118	0.867 (0.725, 1.037)
		AA+AG	145 (32.08)	172 (36.99)	Reference
	Recessive model	AA	14 (3.10)	12 (2.58)	0.637	1.200 (0.561, 2.567)
		AG+GG	438 (96.90)	453 (97.42)	Reference
rs3782499	Genotypes	CC	22 (4.87)	11 (2.37)	0.068	1.994 (0.950, 4.182)
		TC	122 (26.99)	147 (31.61)	0.196	0.827 (0.621, 1.1043
		TT	308 (68.14)	307 (66.02)	Reference
	Alleles	C	166 (18.36)	169 (18.17)	0.916	1.010 (0.833, 1.226)
		T	738 (81.64)	761 (81.83)	Reference
	Dominant model	TT	308 (68.14)	307 (66.02)	0.495	0.938 (0.779, 1.128)
		CC+TC	144 (31.86)	158 (33.98)	Reference
	Recessive model	CC	22 (4.87)	11 (2.37)	**0.042**	2.058 (1.009, 4.194)
		TC+TT	430 (95.13)	454 (97.63)	Reference
*DKK-1*
rs2241529	Genotypes	GG	47 (10.40)	40 (8.60)	0.433	1.205 (0.755, 1.917)
		AG	206 (45.58)	221 (47.53)	0.743	0.956 (0.728, 1.255)
		AA	199 (44.03)	204 (43.87)	Reference
	Alleles	G	300 (33.19)	301 (32.37)	0.708	1.025 (0.899, 1.169)
		A	604 (66.81)	629 (67.63)	Reference
	Dominant model	AA	199 (44.03)	204 (43.87)	0.962	0.997 (0.889, 1.118)
		AG+GG	253 (55.97)	261 (56.13)	Reference
	Recessive model	GG	47 (10.40)	40 (8.60)	0.353	1.209 (0.809, 1.806)
		AA+AG	405 (89.60)	425 (91.40)	Reference
rs1569198	Genotypes	GG	20 (4.42)	22 (4.73)	0.662	0.839 (0.463, 1.630)
		AG	159 (35.18)	182 (39.14)	0.195	0.835 (0.636, 1.096)
		AA	273 (60.40)	261 (56.13)	Reference
	Alleles	G	199 (22.01)	226 (24.30)	0.246	0.906 (0.766, 1.071)
		A	705 (77.99)	704 (75.70)	Reference
	Dominant model	AA	273 (60.40)	261 (56.13)	0.190	1.076 (0.964, 1.201)
		AG+GG	179 (39.60)	204 (43.87)	Reference
	Recessive model	GG	20 (4.42)	22 (4.73)	0.824	0.935 (0.518, 1.690)
		AA+AG	432 (95.58)	443 (95.27)	Reference
*LRP5*						
rs3736228	Genotypes	TT	17 (3.76)	22 (4.73)	0.359	0.737 (0.383, 1.416)
		CT	134 (29.65)	156 (33.55)	0.165	0.819 (0.618, 1.086)
		CC	301 (66.59)	287 (61.72)	Reference
	Alleles	T	168 (18.58)	200 (21.51)	0.118	0.864 (0.719, 1.038)
		C	736 (81.42)	730 (78.49)	Reference
	Dominant model	CC	301 (66.59)	287 (61.72)	0.124	1.079 (0.979, 1.089)
		CT+TT	151 (33.41)	178 (38.28)	Reference
	Recessive model	TT	17 (3.76)	22 (4.73)	0.467	0.795 (0.428, 1.477)
		CC+CT	435 (96.24)	443 (95.27)	Reference
rs556442	Genotypes	GG	33 (7.30)	38 (8.17)	0.416	0.813 (0.494, 0.339)
		GA	168 (37.17)	192 (41.29)	0.152	0.819 (0.624, 1.0776)
		AA	251 (55.53)	235 (50.54)	Reference
	Alleles	G	234 (25.88)	268 (28.82)	0.159	0.898 (0.773, 1.043)
		A	670 (74.12)	662 (71.19)	Reference
	Dominant model	AA	251 (55.53)	235 (50.54)	0.130	1.099 (0.973, 1.241)
		GA+GG	201 (44.47)	230 (49.46)	Reference
	Recessive model	GG	33 (7.30)	38 (8.17)	0.622	0.893 (0.571, 1.398)
		AA+GA	419 (92.70)	427 (91.83)	Reference
*LRP6*						
rs2302685	Genotypes	CC	2 (0.44)	3 (0.65)	0.680	0.686 (0.114, 4.127)
		CT	68 (15.04)	69 (14.84)	0.941	1.014 (0.705, 1.458)
		TT	382 (84.51)	393 (84.52)	Reference
	Alleles	C	72 (7.96)	75 (8.07)	0.937	0.988 (0.724, 1.347)
		T	832 (92.04)	855 (91.93)	Reference
	Dominant model	TT	382 (84.51)	393 (84.52)	0.999	1.000 (0.946, 1.057)
		CC+CT	70 (15.49)	72 (15.48)	Reference
	Recessive model	CC	2 (0.44)	3 (0.65)	0.677	0.686 (0.115, 4.0850
		CT+TT	450 (99.56)	462 (99.35)	Reference
rs11054697	Genotypes	CC	3 (0.66)	4 (0.86)	0.708	0.750 (0.167,3.374)
		TC	68 (15.04)	80 (17.20)	0.367	0.850 (0.597,1.210)
		TT	381 (84.29)	381 (81.94)	Reference
	Alleles	C	74 (8.19)	842 (90.54)	0.335	0.865 (0.644,1.162)
		T	830 (91.81)	88 (9.46)	Reference
	Dominant model	TT	381 (84.29)	381 (81.93)	0.341	1.029 (0.970, 1.091)
		CC+TC	71 (15.71)	84 (18.06)	Reference
	Recessive model	CC	3 (0.66)	4 (0.86)	0.733	0.772 (0.174, 3.428)
		TC+TT	449 (99.34)	461 (99.14)	Reference
rs10743980	Genotypes	TT	31 (6.86)	32 (6.88)	0.871	0.985 (0.568, 1.615)
		TC	157 (34.73)	172 (36.99)	0.466	0.902 (0.685, 1.189)
		CC	264 (58.41)	261 (56.13)	Reference
	Alleles	T	219 (24.23)	236 (25.38)	0.568	0.955 (0.814, 1.120)
		C	685 (75.77)	694 (74.62)	Reference
	Dominant model	CC	264 (58.41)	261 (56.13)	0.486	1.041 (0.930, 1.164)
		TC+TT	188 (41.59)	204 (43.87)	Reference
	Recessive model	TT	31 (6.86)	32 (6.88)	0.989	0.997 (0.619, 1.65)
		CC+TC	421 (93.14)	433 (93.12)	Reference

Bold value means P<0.05.

As illustrated in **Table 1**, there were no significant differences in allele distribution of *SFRP1* rs10088390, rs4736958, rs3242, *WNT3A* rs752107, rs3121310, *CTNNB1* rs2293303, rs1798802, rs4135385, *WIF-1* rs1026024, rs3782499, *DKK-1* rs2241529, rs1569198, *LRP5* rs3736228, rs556442, *LRP6* rs2302685, rs11054697, rs10743980 polymorphism between PTB patients and normal controls (all *P* >0.05). In addition, we did not find any significant association between the genotype distribution of these SNPs and the susceptibility to PTB (all *P* >0.05). It was worth noting that the increased risk of *WIF-1* rs3782499 variant was found to be associated with susceptibility to PTB under recessive model (CC versus TC+TT: *P* = 0.042).

### Association of WNT signaling pathway genes SNPs with several clinical features among with PTB

Among PTB patients, the clinical features of PTB patients were sputum smear-positive (125, 27.65%), pulmonary infection (81, 17.92%), drug resistance (73, 16.15%), fever (69, 15.27%), DILI (64, 14.16%), hypoproteinemia (38, 8.41%), leukopenia (29, 6.42%). We further assessed whether *SFRP1*, *WNT3A*, *CTNNB1*, *WIF-1*, *DKK-1*, *LRP5*, *LRP6* genes variants affected the occurrence of multiple clinical manifestations in PTB patients (**Supplementary Table 1**). The *WNT3A* rs3121310 T allele, *CTNNB1* rs2293303 TT genotype frequencies were significantly decreased in PTB patients with DILI, sputum smear-positive, respectively (*P* = 0.021 P = 0.007). For *WIF-1* gene rs3782499, PTB patients carrying the homozygous TT/CC genotype had a highly significant association with increased frequency of fever, and carrying the C allele had a lower frequency of leukopenia (*P* = 0.032, *P* = 0.048, respectively).

We also found a high frequency of *DKK-1* rs1569198 AG genotype the PTB patients with sputum smear-positive (*P* = 0.049). In *LRP5* gene, the CT genotype frequency of rs3736228 variant was significantly associated with the occurrence of DILI (*P* = 0.040), and the GA genotype frequency of rs556442 variant were significantly related to the risk of fever (*P* = 0.025). In addition, the CT genotype frequency of *LRP6* gene rs2302685 was significantly decreased with hypoproteinemia (*P* = 0.049), and the TC genotype frequency of *LRP6* gene rs10743980 was significantly increased with sputum smear-positive (*P* = 0.007).

### Haplotype analysis

This study firstly detected the haplotypes of *SFRP1*, *WNT3A*, *CTNNB1*, *WIF-1*, *DKK-1*, *LRP5*, *LRP6* genes by SHEsis software, and then analyzed the potential association between these haplotypes and PTB risk. Four main haplotypes (CTA, CTG, GCG, GTG) for *SFRP1*, three main haplotypes (CA, CG, TA) for *WNT3A*, four main haplotypes (CAA, CGA, CGG, TAA) for *CTNNB1*, three main haplotypes (AT, GC, GT) for *WIF-1*, three main haplotypes (AA, GA, GG) for *DKK-1*, three main haplotypes (CA, CG, TG) for *LRP5*, four main haplotypes (CTT, TCT, TTC, TTT) for *LRP6* were detected. The frequency distributions of above haplotypes in PTB group and control group were summarized in **Table 2**.

**Table 2:** Haplotype analysis of WNT pathway genes in PTB patients and controls.

Haplotype	PTB [n(%)]	Controls [n(%)]	*P* value	*OR* (95% CI)
*SFRP1* rs10088390-rs4736958-rs3242				
CTA	26.95 (3.0)	34.30 (3.70)	0.415	0.808(0.484, 1.350)
CTG	516.44 (57.1)	535.24 (57.6)	0.991	0.999(0.827, 1.207)
GCG	285.30 (31.6)	293.36 (31.5)	0.913	1.011(0.829, 1.233)
GTG	46.66 (5.2)	43.93 (4.7)	0.641	1.106(0.724, 1.688)
*WNT3A* rs752107-rs3121310				
CA	142.00 (15.7)	128.35 (13.8)	0.259	1.161(0.896, 1.503)
CG	545.00 (60.3)	567.65 (61.0)	0.692	0.963(0.798, 1.161)
TA	217.00 (24.0)	231.65 (24.9)	0.630	0.949(0.767, 1.174)
*CTNNB1* rs2293303-rs1798802-rs4135385				
CAA	158.80 (17.6)	161.35 (17.3)	0.889	1.017(0.799, 1.295)
CGA	154.24 (17.1)	157.96 (17.0)	0.951	1.008(0.790, 1.286)
CGG	453.23 (50.1)	495.57 (53.3)	0.188	0.884(0.735, 1.062)
TAA	130.44 (14.4)	109.22 (11.7)	0.085	1.270(0.967, 1.668)
*WIF-1* rs1026024-rs3782499				
AT	158.96 (17.6)	183.03 (19.8)	0.228	0.865(0.684, 1.095)
GC	165.96 (18.4)	163.93 (18.2)	0.914	1.013(0.799, 1.284)
GT	579.04 (64.1)	577.07 (6.21)	0.375	1.090(0.901, 1.317)
*DKK-1* rs2241529-rs1569198				
AA	600.60966.4)	626.80 (67.4)	0.692	0.961(0.791, 1.169)
GA	104.40 (11.5)	77.20 (8.3)	**0.019**	1.445(1.060, 1.969)
GG	195.59(21.6)	223.80 (24.1)	0.221	0.873(0.701, 1.086)
*LRP5* rs3736228-rs556442				
CA	667.84 (73.9)	660.92 (71.1)	0.164	1.157(0.942, 1.442)
CG	68.16 (7.5)	69.08 (7.4)	0.921	1.018(0.719, 1.441)
TG	165.84 (18.3)	198.92 (21.4)	0.105	0.827(0.657, 1.041)
*LRP6* rs2302685-rs11054697-rs10743980				
CTT	56.70 (1.4)	56.32 (6.1)	0.860	1.035(0.707, 1.515)
TCT	68.59 (7.6)	85.76 (9.2)	0.200	0.805(0.578, 1.122)
TTC	669.13 (7.4)	673.08 (72.4)	0.476	1.081(0.873, 1.337)
TTT	91.83 (10.2)	93.92 (10.2)	0.983	1.003(0.740, 1.359)

Frequency < 0.03 in both controls & PTB patients has been dropped.

Bold value means P < 0.05.

Comparing the haplotype frequency between the two groups, we found that *DKK-1* GA haplotype frequency was significantly increased in PTB patients than that in controls (*OR* = 1.445, 95% *CI*: 1.060-1.969, *P* = 0.019). However, there was no significant association between other haplotypes frequencies and susceptibility to PTB.

## Discussion

Previous studies had shown that the WNT signaling pathway was crucial in bridging innate and adaptive immunity to MTB infection (11, 16, 17), and the mRNA levels of several WNT signaling pathway genes (CTNNB1, SFRP1 and WNT3A) were significantly increased in PTB patients when compared to controls (3). Functional investigation found that *CTNNB1* gene variation might significantly decreased the promoter transcriptional activity and associated with decreased CTNNB1 mRNA and phosphorylated β-catenin protein level (3), thereby affecting disease susceptibility. Hence, these genes might play important roles in host immune response to MTB infection. In this study, we comprehensively explored the association between 17 SNPs in WNT signaling pathway genes (*SFRP1*, *WNT3A*, *CTNNB1*, *WIF-1*, *DKK-1*, *LRP5*, *LRP6*) and PTB susceptibility, clinical manifestations, providing clues for in-depth understanding of the pathogenesis of PTB.

β-catenin protein, which was encoded by *CTNNB1* gene, played key roles in the coordination of cell adhesion and gene transcription, and aberrant β-catenin was involved in abnormal immune responses to pathogens and disease course, including TB (18, 19). The researchers had also linked *CTNNB1* gene variants to susceptibility and prognosis of some cancers (20, 21). SFRP1 was a mature antagonist of WNT signaling pathway and could block the WNT signaling pathway (22). As a novel inflammatory regulator, SFRP1 might play an indispensable role in the pathogenesis of PTB through regulating the balance between anti-inflammatory cytokines and pro-inflammatory cytokines (23). The results of a previous study showed that *CTNNB1* rs4135385, *SFRP1* rs7832767 variants were associated with PTB risk (24), and another study suggested that *SFRP1* rs4736958, rs7832767 were significantly related to PTB susceptibility and could affect the expression levels of some inflammatory markers in PTB patients (25). These results demonstrated a potential role of these variants in the susceptibility to PTB. Therefore, it was reasonable and necessary to screen other PTB related SNPs in WNT signaling pathway. However, our study did not find any correlation between *SFRP1* rs10088390, rs4736958, rs3242, as well as *CTNNB1* rs2293303, rs1798802, rs4135385 and PTB susceptibility. This inconsistency might be due to the differences in genotyping methods, sample size, *etc* (25). In addition, Hu et al. investigated the roles of several SNPs in *WIF1*, *WNT3A*, *WIF-1*, *DKK-1* genes, and no significant result was found (3). Our results similarly did not support an association between these genetic variants and PTB susceptibility, suggesting that these polymorphisms might not predispose one to this disease. It was worth noting that *WIF-1* gene rs3782499 variant was related to PTB risk under recessive model and *DKK-1* GA haplotype frequency was significantly increased in PTB patients in our study. These results implied that *WIF-1*, *DKK-1* genes variation might be involved in the pathogenesis of PTB, which needed to be verified with multi-ethnic population, large sample size study. As two key genes in WNT signaling pathway, previous studies had demonstrated the genetic association between *LRP5*, *LRP6* and disease susceptibility (26, 27). Our study was the first to analyze the roles of *LRP5* rs3736228, rs556442, *LRP6* rs2302685, rs11054697 and rs10743980 polymorphisms in PTB, and no significant association was observed. Despite these irrelevant results, we could not completely rule out the possibility of these genes as putative PTB-related genes due to imperceptible genotype frequencies or slight effects of these polymorphisms. In addition, the effect of WNT signaling genetic variants on the transcription and protein levels of these genes in PTB patients was also needed to be analyzed in future.

Multiple clinical manifestations of PTB patients, such as fever, drug resistance, DILI, and pulmonary infection, *etc.* might had a certain impact on the rehabilitation and treatment process of patients, and the occurrence of these clinical manifestations was affected by genetic variants (28, 29). Zhao et al. found that PTB patients carrying the CC genotype of *lncRNA AC079767.4* rs12477677 variant were highly significantly correlated with lower frequency of fever (29). Another study suggested that the *lnc-HNF1B-3:1* rs2542670 polymorphism was associated with a higher risk of thrombocytopenia, leukopenia following medication in PTB patients (30). In this study, we investigated whether all candidate SNPs in WNT signaling pathway genes were associated with the clinical manifestations of PTB. Similarly, we observed that *WIF-1* rs3782499, *LRP5* rs556442 variants were related to the occurrence of fever, and *WNT3A* rs3121310, *LRP5* rs3736228 polymorphisms had a significant association with the risk of DILI in PTB patients. Moreover, this study demonstrated a significant correction between several SNPs (*CTNNB1* rs2293303, *DKK-1* rs1569198, *LRP6* rs10743980) and sputum smear-positive in PTB patients. The differences between PTB susceptibility related SNPs and clinical phenotypic related gene variation indicated that PTB development and disease manifestations might be independently affected by different gene SNPs, signaling pathways. While the exact molecular mechanism was still unclear, these results supported that gene variations in WNT signaling pathway could influence host defense responses to MTB infection and lead to the development of different clinical manifestations. This was important to accurately understand the progression of clinical manifestations in PTB patients and contributed to making appropriate treatment.

Although the relationship between WNT signaling pathway genes variants and PTB susceptibility, clinical manifestations was studied in detail, there were still some notable limitations in this study. First, our study did not further analyze the gene-environment or gene-gene interaction because of the uncorrelated-PTB risk among these SNPs. Second, we only selected a small number of SNPs for analysis in the whole WNT signaling pathway, and it was still necessary to study other potential and functional SNPs. Third, all the subjects of this study were only from one hospital, which inevitably resulted in a selection bias.

In conclusion, the present study demonstrated that WNT signaling pathway genes variation might not contribute to PTB susceptibility in Chinese Han population, while several SNPs in *WNT3A*, *CTNNB1*, *WIF-1*, *DKK-1*, *LRP5*, *LRP6* genes were related to the risk of several clinical characteristics of PTB patients. For example, *WNT3A* rs3121310, *CTNNB1* rs2293303 were respectively related DILI, sputum smear-positive, and *WIF-1* rs3782499, *DKK-1* rs1569198 was related to fever, sputum smear-positive. In addition, *LRP5* rs3736228, rs556442 were respectively related to DILI, fever, and *LRP6* rs2302685, rs10743980 were respectively associated with hypoproteinemia, sputum smear-positive. Replication studies with larger sample size, different ethnic groups were required to verify and explore the genetic mechanisms of WNT signaling pathway in PTB development.

## Data availability statement

The data presented in the study are deposited in the dbSNP (1063448). Further inquiries can be directed to the corresponding authors.

## Ethics statement

The studies involving human participants were reviewed and approved by the Ethics Committee of Anhui Chest Hospital. The patients/participants provided their written informed consent to participate in this study.

## Author contributions

YL, HW, and QH designed the study. QH and T-PZ conducted the experiment. QH and C-CW performed the statistical analyses. HW participated in sample collection. QH drafted the manuscript. Y-GL and C-MZ contributed to the manuscript revision. All authors approved the final submitted version.
