# Supplementary material for: Clinical relevance of genetic polymorphisms in WNT signaling pathway (SFRP1, WNT3A, CTNNB1, WIF-1, DKK-1, LRP5, LRP6) on pulmonary tuberculosis in a Chinese population
Source: Front Immunol. 2022 Dec 7;13:1011700. doi: 10.3389/fimmu.2022.1011700 (PMC9768481; doi:10.3389/fimmu.2022.1011700)
Supplement: Supplementary file 1 [file Table_1.docx]

**Supplementary Table 1** Association of WNT pathway genes polymorphisms and the clinical features in PTB patients

| SNP | Allele | Clinical features | Group | Genotypes n (%) | | | *P* value | Alleles n (%) | | *P* value |
| --- | --- | --- | --- | --- | --- | --- | --- | --- | --- | --- |
|  | (M/m) |  |  | MM | Mm | mm |  | M | m |  |
| *SFRP1* | | | | | | | | | | |
| rs10088390 | C/G | fever | + | 27(39.13) | 35(50.72) | 7(10.14) | 0.492 | 89(64.49) | 49(35.51) | 0.354 |
|  |  |  | - | 139(36.29) | 184(48.04) | 60(15.67) |  | 462(60.31) | 304(39.69) |  |
|  |  | drug resistance | + | 46(63.01) | 24(32.88) | 3(4.11) | 0.506 | 116(79.45) | 30(20.55) | 0.257 |
|  |  |  | - | 218(57.52) | 133(35.09) | 28(7.39) |  | 569(75.07) | 189(24.93) |  |
|  |  | DILI | + | 19(29.69) | 38(59.38) | 7(10.94) | 0.164 | 76(59.38) | 52(40.63) | 0.693 |
|  |  |  | - | 147(37.89) | 181(46.65) | 60(15.46) |  | 475(61.21) | 301(38.79) |  |
|  |  | pulmonary infection | + | 25(30.86) | 40(49.38) | 16(19.75) | 0.278 | 90(55.56) | 72(44.44) | 0.120 |
|  |  |  | - | 141(38.01) | 179(48.25) | 51(13.75) |  | 461(62.13) | 281(37.87) |  |
|  |  | hypoproteinemia | + | 12(31.58) | 24(63.16) | 2(5.26) | 0.095 | 48(63.16) | 28(36.84) | 0.680 |
|  |  |  | - | 154(37.2) | 195(47.1) | 65(15.7) |  | 503(60.75) | 325(39.25) |  |
|  |  | leukopenia | + | 10(34.48) | 14(48.28) | 5(17.24) | 0.921 | 34(58.62) | 24(41.38) | 0.707 |
|  |  |  | - | 156(36.88) | 205(48.46) | 62(14.66) |  | 517(61.11) | 329(38.89) |  |
|  |  | sputum smear-positive | + | 47(37.6) | 62(49.6) | 16(12.8) | 0.563 | 156(62.4) | 94(37.6) | 0.383 |
|  |  |  | - | 102(35.29) | 138(47.75) | 49(16.96) |  | 342(59.17) | 236(40.83) |  |
| rs4736958 | T/C | fever | + | 31(44.93) | 33(47.83) | 5(7.25) | 0.543 | 95(68.84) | 43(31.16) | 0.733 |
|  |  |  | - | 176(45.95) | 164(42.82) | 43(11.23) |  | 516(67.36) | 250(32.64) |  |
|  |  | drug resistance | + | 34(46.58) | 31(42.47) | 8(10.96) | 0.977 | 99(67.81) | 47(32.19) | 0.951 |
|  |  |  | - | 173(45.65) | 166(43.8) | 40(10.55) |  | 512(67.55) | 246(32.45) |  |
|  |  | DILI | + | 27(42.19) | 34(53.13) | 3(4.69) | 0.120 | 88(68.75) | 40(31.25) | 0.762 |
|  |  |  | - | 180(46.39) | 163(42.01) | 45(11.6) |  | 523(67.4) | 253(32.6) |  |
|  |  | pulmonary infection | + | 28(34.57) | 44(54.32) | 9(11.11) | 0.069 | 100(61.73) | 62(38.27) | 0.079 |
|  |  |  | - | 179(48.25) | 153(41.24) | 39(10.51) |  | 511(68.87) | 231(31.13) |  |
|  |  | hypoproteinemia | + | 16(42.11) | 20(52.63) | 2(5.26) | 0.364 | 52(68.42) | 24(31.58) | 0.871 |
|  |  |  | - | 191(46.14) | 177(42.75) | 46(11.11) |  | 559(67.51) | 269(32.49) |  |
|  |  | leukopenia | + | 14(48.28) | 12(41.38) | 3(10.34) | 0.962 | 40(68.97) | 18(31.03) | 0.817 |
|  |  |  | - | 193(45.63) | 185(43.74) | 45(10.64) |  | 571(67.49) | 275(32.51) |  |
|  |  | sputum smear-positive | + | 62(49.6) | 51(40.8) | 12(9.6) | 0.427 | 175(70) | 75(30) | 0.197 |
|  |  |  | - | 124(42.91) | 130(44.98) | 35(12.11) |  | 378(65.4) | 200(34.6) |  |
| rs3242 | G/A | fever | + | 66(95.65) | 3(4.35) | 0(0) | 0.227 | 135(97.83) | 3(2.17) | 0.074 |
|  |  |  | - | 341(89.03) | 39(10.18) | 3(0.78) |  | 721(94.13) | 45(5.87) |  |
|  |  | drug resistance | + | 70(95.89) | 2(2.74) | 1(1.37) | 0.082 | 142(97.26) | 4(2.74) | 0.130 |
|  |  |  | - | 337(88.92) | 40(10.55) | 2(0.53) |  | 714(94.2) | 44(5.8) |  |
|  |  | DILI | + | 58(90.63) | 5(7.81) | 1(1.56) | 0.581 | 121(94.53) | 7(5.47) | 0.931 |
|  |  |  | - | 349(89.95) | 37(9.54) | 2(0.52) |  | 735(94.72) | 41(5.28) |  |
|  |  | pulmonary infection | + | 76(93.83) | 5(6.17) | 0(0) | 0.398 | 157(96.91) | 5(3.09) | 0.164 |
|  |  |  | - | 331(89.22) | 37(9.97) | 3(0.81) |  | 699(94.2) | 43(5.8) |  |
|  |  | hypoproteinemia | + | 36(94.74) | 2(5.26) | 0(0) | 0.577 | 74(97.37) | 2(2.63) | 0.277 |
|  |  |  | - | 371(89.61) | 40(9.66) | 3(0.72) |  | 782(94.44) | 46(5.56) |  |
|  |  | leukopenia | + | 26(89.66) | 2(6.9) | 1(3.45) | 0.149 | 54(93.1) | 4(6.9) | 0.577 |
|  |  |  | - | 381(90.07) | 40(9.46) | 2(0.47) |  | 802(94.8) | 44(5.2) |  |
|  |  | sputum smear-positive | + | 111(88.8) | 13(10.4) | 1(0.8) | 0.844 | 235(94) | 15(6) | 0.563 |
|  |  |  | - | 262(90.66) | 25(8.65) | 2(0.69) |  | 549(94.98) | 29(5.02) |  |
| *WNT3A* | | | | | | | | | | |
| rs752107 | C/T | fever | + | 41(59.42) | 24(34.78) | 4(5.80) | 0.971 | 106(76.81) | 32(23.19) | 0.807 |
|  |  |  | - | 222(57.96) | 137(35.77) | 24(6.27) |  | 581(75.85) | 185(24.15) |  |
|  |  | drug resistance | + | 48(65.75) | 23(31.51) | 2(2.74) | 0.229 | 119(81.51) | 27(18.49) | 0.089 |
|  |  |  | - | 215(56.73) | 138(36.41) | 26(6.86) |  | 568(74.93) | 190(25.07) |  |
|  |  | DILI | + | 42(65.63) | 21(32.81) | 1(1.56) | 0.178 | 105(82.03) | 23(17.97) | 0.084 |
|  |  |  | - | 221(56.96) | 140(36.08) | 27(6.96) |  | 582(75) | 194(25) |  |
|  |  | pulmonary infection | + | 48(59.26) | 30(37.04) | 3(3.70) | 0.588 | 126(77.78) | 36(22.22) | 0.558 |
|  |  |  | - | 215(57.95) | 131(35.31) | 25(6.74) |  | 561(75.61) | 181(24.39) |  |
|  |  | hypoproteinemia | + | 23(60.53) | 12(31.58) | 3(7.89) | 0.809 | 58(76.32) | 18(23.68) | 0.946 |
|  |  |  | - | 240(57.97) | 149(35.99) | 25(6.04) |  | 629(75.97) | 199(24.03) |  |
|  |  | leukopenia | + | 16(55.17) | 13(44.83) | 0(0) | 0.259 | 45(77.59) | 13(22.41) | 0.769 |
|  |  |  | - | 247(58.39) | 148(34.99) | 28(6.62) |  | 642(75.89) | 204(24.11) |  |
|  |  | sputum smear-positive | + | 70(560.00) | 46(36.80) | 9(7.20) | 0.739 | 186(74.40) | 64(25.60) | 0.454 |
|  |  |  | - | 171(59.17) | 102(35.29) | 16(5.54) |  | 444(76.82) | 134(23.18) |  |
| rs3121310 | C/T | fever | + | 25(36.23) | 36(52.17) | 8(11.59) | 0.474 | 86(62.32) | 52(37.68) | 0.596 |
|  |  |  | - | 141(36.81) | 177(46.21) | 65(16.97) |  | 459(59.92) | 307(40.08) |  |
|  |  | drug resistance | + | 33(45.21) | 29(39.73) | 11(15.07) | 0.249 | 95(65.07) | 51(34.93) | 0.197 |
|  |  |  | - | 133(35.09) | 184(48.55) | 62(16.36) |  | 450(59.37) | 308(40.63) |  |
|  |  | DILI | + | 30(46.88) | 29(45.31) | 5(7.81) | 0.069 | 89(69.53) | 39(30.47) | **0.021** |
|  |  |  | - | 136(35.05) | 184(47.42) | 68(17.53) |  | 456(58.76) | 320(41.24) |  |
|  |  | pulmonary infection | + | 32(39.51) | 39(48.15) | 10(12.35) | 0.573 | 103(63.58) | 59(36.42) | 0.344 |
|  |  |  | - | 134(36.12) | 174(46.9) | 63(16.98) |  | 442(59.57) | 300(40.43) |  |
|  |  | hypoproteinemia | + | 15(39.47) | 17(44.74) | 6(15.79) | 0.933 | 47(61.84) | 29(38.16) | 0.772 |
|  |  |  | - | 151(36.47) | 196(47.34) | 67(16.18) |  | 498(60.14) | 330(39.86) |  |
|  |  | leukopenia | + | 12(41.38) | 11(37.93) | 6(20.69) | 0.567 | 35(60.34) | 23(39.66) | 0.993 |
|  |  |  | - | 154(36.41) | 202(47.75) | 67(15.84) |  | 510(60.28) | 336(39.72) |  |
|  |  | sputum smear-positive | + | 41(32.80) | 63(50.40) | 21(16.80) | 0.513 | 145(58.00) | 105(42.00) | 0.332 |
|  |  |  | - | 112(38.75) | 132(45.67) | 45(15.57) |  | 356(61.59) | 222(38.41) |  |
| *CTNNB1* | | | | | | | | | | |
| rs2293303 | C/T | fever | + | 51(73.91) | 18(26.09) | 0(0) | 0.321 | 120(86.96) | 18(13.04) | 0.523 |
|  |  |  | - | 279(72.85) | 92(24.02) | 12(3.13) |  | 650(84.86) | 116(15.14) |  |
|  |  | drug resistance | + | 56(76.71) | 17(23.29) | 0(0) | 0.284 | 129(88.36) | 17(11.64) | 0.238 |
|  |  |  | - | 274(72.3) | 93(24.54) | 12(3.17) |  | 641(84.56) | 117(15.44) |  |
|  |  | DILI | + | 47(73.44) | 16(25) | 1(1.56) | 0.839 | 110(85.94) | 18(14.06) | 0.794 |
|  |  |  | - | 283(72.94) | 94(24.23) | 11(2.84) |  | 660(85.05) | 116(14.95) |  |
|  |  | pulmonary infection | + | 54(66.67) | 23(28.4) | 4(4.94) | 0.207 | 131(80.86) | 31(19.14) | 0.088 |
|  |  |  | - | 276(74.39) | 87(23.45) | 8(2.16) |  | 639(86.12) | 103(13.88) |  |
|  |  | hypoproteinemia | + | 29(76.32) | 8(21.05) | 1(2.63) | 0.884 | 66(86.84) | 10(13.16) | 0.669 |
|  |  |  | - | 301(72.71) | 102(24.64) | 11(2.66) |  | 704(85.02) | 124(14.98) |  |
|  |  | leukopenia | + | 22(75.86) | 6(20.69) | 1(3.45) | 0.871 | 50(86.21) | 8(13.79) | 0.820 |
|  |  |  | - | 308(72.81) | 104(24.59) | 11(2.6) |  | 720(85.11) | 126(14.89) |  |
|  |  | sputum smear-positive | + | 85(68.00) | 40(32.00) | 0(0) | **0.007** | 210(84.00) | 40(16.00) | 0.380 |
|  |  |  | - | 220(76.12) | 59(20.42) | 10(3.46) |  | 499(86.33) | 79(13.67) |  |
| rs1798802 | G/A | fever | + | 25(36.23) | 38(55.07) | 6(8.70) | 0.266 | 88(63.77) | 50(36.23) | 0.298 |
|  |  |  | - | 176(45.95) | 171(44.65) | 36(9.40) |  | 523(68.28) | 243(31.72) |  |
|  |  | drug resistance | + | 33(45.21) | 35(47.95) | 5(6.85) | 0.732 | 101(69.18) | 45(30.82) | 0.654 |
|  |  |  | - | 168(44.33) | 174(45.91) | 37(9.76) |  | 510(67.28) | 248(32.72) |  |
|  |  | DILI | + | 33(51.56) | 22(34.38) | 9(14.06) | 0.085 | 88(68.75) | 40(31.25) | 0.762 |
|  |  |  | - | 168(43.30) | 187(48.20) | 33(8.51) |  | 523(67.40) | 253(32.60) |  |
|  |  | pulmonary infection | + | 30(37.04) | 40(49.38) | 11(13.58) | 0.184 | 100(61.73) | 62(38.27) | 0.079 |
|  |  |  | - | 171(46.09) | 169(45.55) | 31(8.36) |  | 511(68.87) | 231(31.13) |  |
|  |  | hypoproteinemia | + | 16(42.11) | 17(44.74) | 5(13.16) | 0.691 | 49(64.47) | 27(35.53) | 0.544 |
|  |  |  | - | 185(44.69) | 192(46.38) | 37(8.94) |  | 562(67.87) | 266(32.13) |  |
|  |  | leukopenia | + | 12(41.38) | 12(41.38) | 5(17.24) | 0.312 | 36(62.07) | 22(37.93) | 0.353 |
|  |  |  | - | 189(44.68) | 197(46.57) | 37(8.75) |  | 575(67.97) | 271(32.03) |  |
|  |  | sputum smear-positive | + | 54(43.20) | 59(47.20) | 12(9.60) | 0.843 | 167(66.80) | 83(33.20) | 0.628 |
|  |  |  | - | 130(44.98) | 136(47.06) | 23(7.96) |  | 396(68.51) | 182(31.49) |  |
| rs4135385 | G/A | fever | + | 17(24.64) | 39(56.52) | 13(18.84) | 0.538 | 73(52.90) | 65(47.10) | 0.709 |
|  |  |  | - | 96(25.07) | 200(52.22) | 87(22.72) |  | 392(51.17) | 374(48.83) |  |
|  |  | drug resistance | + | 17(23.29) | 41(56.16) | 15(20.55) | 0.804 | 75(51.37) | 71(48.63) | 0.829 |
|  |  |  | - | 92(24.27) | 198(52.24) | 89(23.48) |  | 382(50.40) | 376(49.60) |  |
|  |  | DILI | + | 20(31.25) | 30(46.88) | 14(21.88) | 0.346 | 70(54.69) | 58(45.31) | 0.339 |
|  |  |  | - | 90(23.2) | 209(53.87) | 89(22.94) |  | 389(50.13) | 387(49.87) |  |
|  |  | pulmonary infection | + | 22(27.16) | 42(51.85) | 17(20.99) | 0.560 | 86(53.09) | 76(46.91) | 0.688 |
|  |  |  | - | 92(24.8) | 197(53.1) | 82(22.1) |  | 381(51.35) | 361(48.65) |  |
|  |  | hypoproteinemia | + | 7(18.42) | 24(63.16) | 7(18.42) | 0.413 | 38(50) | 38(50) | 0.920 |
|  |  |  | - | 102(24.64) | 215(51.93) | 97(23.43) |  | 419(50.6) | 409(49.4) |  |
|  |  | leukopenia | + | 7(24.14) | 18(62.07) | 4(13.79) | 0.390 | 32(55.17) | 26(44.83) | 0.533 |
|  |  |  | - | 105(24.82) | 221(52.25) | 97(22.93) |  | 431(50.95) | 415(49.05) |  |
|  |  | sputum smear-positive | + | 29(23.2) | 72(57.6) | 24(19.2) | 0.297 | 130(52) | 120(48) | 0.947 |
|  |  |  | - | 76(26.3) | 150(51.9) | 63(21.8) |  | 302(52.25) | 276(47.75) |  |
| *WIF-1* | | | | | | | | | | |
| rs1026024 | G/A | fever | + | 48(69.57) | 21(30.43) | 0(0) | 0.271 | 117(84.78) | 21(15.22) | 0.427 |
|  |  |  | - | 259(67.62) | 110(28.72) | 14(3.66) |  | 628(81.98) | 138(18.02) |  |
|  |  | drug resistance | + | 56(76.71) | 14(19.18) | 3(4.11) | 0.125 | 126(86.3) | 20(13.7) | 0.178 |
|  |  |  | - | 251(66.23) | 117(30.87) | 11(2.9) |  | 619(81.66) | 139(18.34) |  |
|  |  | DILI | + | 44(68.75) | 18(28.13) | 2(3.13) | 0.987 | 106(82.81) | 22(17.19) | 0.898 |
|  |  |  | - | 263(67.78) | 113(29.12) | 12(3.09) |  | 639(82.35) | 137(17.65) |  |
|  |  | pulmonary infection | + | 54(66.67) | 27(33.33) | 0(0) | 0.155 | 135(83.33) | 27(16.67) | 0.734 |
|  |  |  | - | 253(68.19) | 104(28.03) | 14(3.77) |  | 610(82.21) | 132(17.79) |  |
|  |  | hypoproteinemia | + | 26(68.42) | 11(28.95) | 1(2.63) | 0.985 | 63(82.89) | 13(17.11) | 0.908 |
|  |  |  | - | 281(67.87) | 120(28.99) | 13(3.14) |  | 682(82.37) | 146(17.63) |  |
|  |  | leukopenia | + | 16(55.17) | 12(41.38) | 1(3.45) | 0.302 | 44(75.86) | 14(24.14) | 0.176 |
|  |  |  | - | 291(68.79) | 119(28.13) | 13(3.07) |  | 701(82.86) | 145(17.14) |  |
|  |  | sputum smear-positive | + | 89(71.2) | 33(26.4) | 3(2.4) | 0.533 | 211(84.4) | 39(15.6) | 0.261 |
|  |  |  | - | 191(66.09) | 87(30.1) | 11(3.81) |  | 469(81.14) | 109(18.86) |  |
| rs3782499 | T/C | fever | + | 54(78.26) | 10(14.49) | 5(7.25) | **0.032** | 118(85.51) | 20(14.49) | 0.202 |
|  |  |  | - | 254(66.32) | 112(29.24) | 17(4.44) |  | 620(80.94) | 146(19.06) |  |
|  |  | drug resistance | + | 49(67.12) | 20(27.4) | 4(5.48) | 0.958 | 118(80.82) | 28(19.18) | 0.781 |
|  |  |  | - | 259(68.34) | 102(26.91) | 18(4.75) |  | 620(81.79) | 138(18.21) |  |
|  |  | DILI | + | 49(76.56) | 14(21.88) | 1(1.56) | 0.205 | 112(87.5) | 16(12.5) | 0.064 |
|  |  |  | - | 259(66.75) | 108(27.84) | 21(5.41) |  | 626(80.67) | 150(19.33) |  |
|  |  | pulmonary infection | + | 51(62.96) | 24(29.63) | 6(7.41) | 0.377 | 126(77.78) | 36(22.22) | 0.161 |
|  |  |  | - | 257(69.27) | 98(26.42) | 16(4.31) |  | 612(82.48) | 130(17.52) |  |
|  |  | hypoproteinemia | + | 24(63.16) | 11(28.95) | 3(7.89) | 0.609 | 59(77.63) | 17(22.37) | 0.346 |
|  |  |  | - | 284(68.6) | 111(26.81) | 19(4.59) |  | 679(82) | 149(18) |  |
|  |  | leukopenia | + | 24(82.76) | 5(17.24) | 0(0) | 0.168 | 53(91.38) | 5(8.62) | **0.048** |
|  |  |  | - | 284(67.14) | 117(27.66) | 22(5.2) |  | 685(80.97) | 161(19.03) |  |
|  |  | sputum smear-positive | + | 88(70.4) | 30(24) | 7(5.6) | 0.536 | 206(82.4) | 44(17.6) | 0.755 |
|  |  |  | - | 194(67.13) | 83(28.72) | 12(4.15) |  | 471(81.49) | 107(18.51) |  |
| *DKK-1* | | | | | | | | | | |
| rs2241529 | A/G | fever | + | 34(49.28) | 31(44.93) | 4(5.8) | 0.337 | 99(71.74) | 39(28.26) | 0.182 |
|  |  |  | - | 165(43.08) | 175(45.69) | 43(11.23) |  | 505(65.93) | 261(34.07) |  |
|  |  | drug resistance | + | 29(39.73) | 35(47.95) | 9(12.33) | 0.675 | 93(63.7) | 53(36.3) | 0.383 |
|  |  |  | - | 170(44.85) | 171(45.12) | 38(10.03) |  | 511(67.41) | 247(32.59) |  |
|  |  | DILI | + | 25(39.06) | 27(42.19) | 12(18.75) | 0.061 | 77(60.16) | 51(39.84) | 0.084 |
|  |  |  | - | 174(44.85) | 179(46.13) | 35(9.02) |  | 527(67.91) | 249(32.09) |  |
|  |  | pulmonary infection | + | 34(41.98) | 38(46.91) | 9(11.11) | 0.913 | 106(65.43) | 56(34.57) | 0.680 |
|  |  |  | - | 165(44.47) | 168(45.28) | 38(10.24) |  | 498(67.12) | 244(32.88) |  |
|  |  | hypoproteinemia | + | 14(36.84) | 20(52.63) | 4(10.53) | 0.625 | 48(63.16) | 28(36.84) | 0.479 |
|  |  |  | - | 185(44.69) | 186(44.93) | 43(10.39) |  | 556(67.15) | 272(32.85) |  |
|  |  | leukopenia | + | 11(37.93) | 15(51.72) | 3(10.34) | 0.772 | 37(63.79) | 21(36.21) | 0.614 |
|  |  |  | - | 188(44.44) | 191(45.15) | 44(10.4) |  | 567(67.02) | 279(32.98) |  |
|  |  | sputum smear-positive | + | 49(39.2) | 66(52.8) | 10(8) | 0.095 | 164(65.6) | 86(34.4) | 0.778 |
|  |  |  | - | 132(45.67) | 121(41.87) | 36(12.46) |  | 385(66.61) | 193(33.39) |  |
| rs1569198 | A/G | fever | + | 42(60.87) | 25(36.23) | 2(2.9) | 0.796 | 109(78.99) | 29(21.01) | 0.758 |
|  |  |  | - | 231(60.31) | 134(34.99) | 18(4.7) |  | 596(77.81) | 170(22.19) |  |
|  |  | drug resistance | + | 39(53.42) | 30(41.1) | 4(5.48) | 0.409 | 108(73.97) | 38(26.03) | 0.201 |
|  |  |  | - | 234(61.74) | 129(34.04) | 16(4.22) |  | 597(78.76) | 161(21.24) |  |
|  |  | DILI | + | 41(64.06) | 17(26.56) | 6(9.38) | 0.053 | 99(77.34) | 29(22.66) | 0.850 |
|  |  |  | - | 232(59.79) | 142(36.6) | 14(3.61) |  | 606(78.09) | 170(21.91) |  |
|  |  | pulmonary infection | + | 46(56.79) | 33(40.74) | 2(2.47) | 0.380 | 125(77.16) | 37(22.84) | 0.779 |
|  |  |  | - | 227(61.19) | 126(33.96) | 18(4.85) |  | 580(78.17) | 162(21.83) |  |
|  |  | hypoproteinemia | + | 21(55.26) | 16(42.11) | 1(2.63) | 0.592 | 58(76.32) | 18(23.68) | 0.713 |
|  |  |  | - | 252(60.87) | 143(34.54) | 19(4.59) |  | 647(78.14) | 181(21.86) |  |
|  |  | leukopenia | + | 18(62.07) | 9(31.03) | 2(6.9) | 0.743 | 45(77.59) | 13(22.41) | 0.939 |
|  |  |  | - | 255(60.28) | 150(35.46) | 18(4.26) |  | 660(78.01) | 186(21.99) |  |
|  |  | sputum smear-positive | + | 67(53.6) | 54(43.2) | 4(3.2) | **0.049** | 188(75.2) | 62(24.8) | 0.241 |
|  |  |  | - | 183(63.32) | 90(31.14) | 16(5.54) |  | 456(78.89) | 122(21.11) |  |
| *LRP5* | | | | | | | | | | |
| rs3736228 | C/T | fever | + | 42(60.87) | 26(37.68) | 1(1.45) | 0.189 | 110(79.71) | 28(20.29) | 0.576 |
|  |  |  | - | 259(67.62) | 108(28.2) | 16(4.18) |  | 626(81.72) | 140(18.28) |  |
|  |  | drug resistance | + | 48(65.75) | 24(32.88) | 1(1.37) | 0.441 | 120(82.19) | 26(17.81) | 0.792 |
|  |  |  | - | 253(66.75) | 110(29.02) | 16(4.22) |  | 616(81.27) | 142(18.73) |  |
|  |  | DILI | + | 38(59.38) | 26(40.63) | 0(0) | **0.040** | 102(79.69) | 26(20.31) | 0.587 |
|  |  |  | - | 263(67.78) | 108(27.84) | 17(4.38) |  | 634(81.7) | 142(18.3) |  |
|  |  | pulmonary infection | + | 54(66.67) | 25(30.86) | 2(2.47) | 0.784 | 133(82.1) | 29(17.9) | 0.805 |
|  |  |  | - | 247(66.58) | 109(29.38) | 15(4.04) |  | 603(81.27) | 139(18.73) |  |
|  |  | hypoproteinemia | + | 22(57.89) | 13(34.21) | 3(7.89) | 0.266 | 57(75) | 19(25) | 0.133 |
|  |  |  | - | 279(67.39) | 121(29.23) | 14(3.38) |  | 679(82) | 149(18) |  |
|  |  | leukopenia | + | 19(65.52) | 10(34.48) | 0(0) | 0.493 | 48(82.76) | 10(17.24) | 0.786 |
|  |  |  | - | 282(66.67) | 124(29.31) | 17(4.02) |  | 688(81.32) | 158(18.68) |  |
|  |  | sputum smear-positive | + | 87(69.6) | 36(28.8) | 2(1.6) | 0.281 | 210(84) | 40(16) | 0.249 |
|  |  |  | - | 191(66.09) | 84(29.07) | 14(4.84) |  | 466(80.62) | 112(19.38) |  |
| rs556442 | A/G | fever | - | 32(46.38) | 35(50.72) | 2(2.9) | **0.025** | 99(71.74) | 39(28.26) | 0.489 |
|  |  |  | + | 219(57.18) | 133(34.73) | 31(8.09) |  | 571(74.54) | 195(25.46) |  |
|  |  | drug resistance | - | 39(53.42) | 32(43.84) | 2(2.74) | 0.166 | 110(75.34) | 36(24.66) | 0.712 |
|  |  |  | + | 212(55.94) | 136(35.88) | 31(8.18) |  | 560(73.88) | 198(26.12) |  |
|  |  | DILI | - | 35(54.69) | 28(43.75) | 1(1.56) | 0.120 | 98(76.56) | 30(23.44) | 0.495 |
|  |  |  | + | 216(55.67) | 140(36.08) | 32(8.25) |  | 572(73.71) | 204(26.29) |  |
|  |  | pulmonary infection | - | 42(51.85) | 36(44.44) | 3(3.7) | 0.183 | 120(74.07) | 42(25.93) | 0.990 |
|  |  |  | + | 209(56.33) | 132(35.58) | 30(8.09) |  | 550(74.12) | 192(25.88) |  |
|  |  | hypoproteinemia | - | 20(52.63) | 14(36.84) | 4(10.53) | 0.721 | 54(71.05) | 22(28.95) | 0.524 |
|  |  |  | + | 231(55.8) | 154(37.2) | 29(7) |  | 616(74.4) | 212(25.6) |  |
|  |  | leukopenia | - | 18(62.07) | 9(31.03) | 2(6.9) | 0.756 | 45(77.59) | 13(22.41) | 0.533 |
|  |  |  | + | 233(55.08) | 159(37.59) | 31(7.33) |  | 625(73.88) | 221(26.12) |  |
|  |  | sputum smear-positive | - | 74(59.2) | 47(37.6) | 4(3.2) | 0.090 | 195(78) | 55(22) | 0.096 |
|  |  |  | + | 157(54.33) | 105(36.33) | 27(9.34) |  | 419(72.49) | 159(27.51) |  |
| *LRP6* | | | | | | | | | | |
| rs2302685 | T/C | fever | + | 59(85.51) | 10(14.49) | 0(0) | 0.824 | 128(92.75) | 10(7.25) | 0.735 |
|  |  |  | - | 323(84.33) | 58(15.14) | 2(0.52) |  | 704(91.91) | 62(8.09) |  |
|  |  | drug resistance | + | 62(84.93) | 11(15.07) | 0(0) | 0.824 | 135(92.47) | 11(7.53) | 0.834 |
|  |  |  | - | 320(84.43) | 57(15.04) | 2(0.53) |  | 697(91.95) | 61(8.05) |  |
|  |  | DILI | + | 50(78.13) | 13(20.31) | 1(1.56) | 0.146 | 113(88.28) | 15(11.72) | 0.090 |
|  |  |  | - | 332(85.57) | 55(14.18) | 1(0.26) |  | 719(92.65) | 57(7.35) |  |
|  |  | pulmonary infection | + | 72(88.89) | 9(11.11) | 0(0) | 0.433 | 153(94.44) | 9(5.56) | 0.211 |
|  |  |  | - | 310(83.56) | 59(15.9) | 2(0.54) |  | 679(91.51) | 63(8.49) |  |
|  |  | hypoproteinemia | + | 34(89.47) | 3(7.89) | 1(2.63) | **0.049** | 71(93.42) | 5(6.58) | 0.641 |
|  |  |  | - | 348(84.06) | 65(15.7) | 1(0.24) |  | 761(91.91) | 67(8.09) |  |
|  |  | leukopenia | + | 22(75.86) | 7(24.14) | 0(0) | 0.347 | 51(87.93) | 7(12.07) | 0.233 |
|  |  |  | - | 360(85.11) | 61(14.42) | 2(0.47) |  | 781(92.32) | 65(7.68) |  |
|  |  | sputum smear-positive | + | 103(82.4) | 21(16.8) | 1(0.8) | 0.603 | 227(90.8) | 23(9.2) | 0.342 |
|  |  |  | - | 248(85.81) | 40(13.84) | 1(0.35) |  | 536(92.73) | 42(7.27) |  |
| rs11054697 | T/C | fever | + | 58(84.06) | 11(15.94) | 0(0) | 0.747 | 127(92.03) | 11(7.97) | 0.920 |
|  |  |  | - | 323(84.33) | 57(14.88) | 3(0.78) |  | 703(91.78) | 63(8.22) |  |
|  |  | drug resistance | + | 63(86.3) | 10(13.7) | 0(0) | 0.696 | 136(93.15) | 10(6.85) | 0.520 |
|  |  |  | - | 318(83.91) | 58(15.3) | 3(0.79) |  | 694(91.56) | 64(8.44) |  |
|  |  | DILI | + | 55(85.94) | 8(12.5) | 1(1.56) | 0.535 | 118(92.19) | 10(7.81) | 0.868 |
|  |  |  | - | 326(84.02) | 60(15.46) | 2(0.52) |  | 712(91.75) | 64(8.25) |  |
|  |  | pulmonary infection | + | 69(85.19) | 12(14.81) | 0(0) | 0.716 | 150(92.59) | 12(7.41) | 0.690 |
|  |  |  | - | 312(84.1) | 56(15.09) | 3(0.81) |  | 680(91.64) | 62(8.36) |  |
|  |  | hypoproteinemia | + | 34(89.47) | 4(10.53) | 0(0) | 0.616 | 72(94.74) | 4(5.26) | 0.331 |
|  |  |  | - | 347(83.82) | 64(15.46) | 3(0.72) |  | 758(91.55) | 70(8.45) |  |
|  |  | leukopenia | + | 24(82.76) | 5(17.24) | 0(0) | 0.855 | 53(91.38) | 5(8.62) | 0.901 |
|  |  |  | - | 357(84.4) | 63(14.89) | 3(0.71) |  | 777(91.84) | 69(8.16) |  |
|  |  | sputum smear-positive | + | 105(84) | 20(16) | 0(0) | 0.514 | 230(92) | 20(8) | 0.757 |
|  |  |  | - | 242(83.74) | 44(15.22) | 3(1.04) |  | 528(91.35) | 50(8.65) |  |
| rs10743980 | C/T | fever | + | 39(56.52) | 27(39.13) | 3(4.35) | 0.533 | 105(76.09) | 33(23.91) | 0.926 |
|  |  |  | - | 225(58.75) | 130(33.94) | 28(7.31) |  | 580(75.72) | 186(24.28) |  |
|  |  | drug resistance | + | 46(63.01) | 24(32.88) | 3(4.11) | 0.506 | 116(79.45) | 30(20.55) | 0.257 |
|  |  |  | - | 218(57.52) | 133(35.09) | 28(7.39) |  | 569(75.07) | 189(24.93) |  |
|  |  | DILI | + | 35(54.69) | 22(34.38) | 7(10.94) | 0.370 | 92(71.88) | 36(28.13) | 0.266 |
|  |  |  | - | 229(59.02) | 135(34.79) | 24(6.19) |  | 593(76.42) | 183(23.58) |  |
|  |  | pulmonary infection | + | 46(56.79) | 29(35.8) | 6(7.41) | 0.942 | 121(74.69) | 41(25.31) | 0.723 |
|  |  |  | - | 218(58.76) | 128(34.5) | 25(6.74) |  | 564(76.01) | 178(23.99) |  |
|  |  | hypoproteinemia | + | 19(50) | 18(47.37) | 1(2.63) | 0.175 | 56(73.68) | 20(26.32) | 0.657 |
|  |  |  | - | 245(59.18) | 139(33.57) | 30(7.25) |  | 629(75.97) | 199(24.03) |  |
|  |  | sputum smear-positive | + | 15(51.72) | 10(34.48) | 4(13.79) | 0.300 | 40(68.97) | 18(31.03) | 0.211 |
|  |  |  | - | 249(58.87) | 147(34.75) | 27(6.38) |  | 645(76.24) | 201(23.76) |  |
|  |  | sputum smear-positive | + | 67(53.6) | 55(44.00) | 3(2.4) | **0.007** | 189(75.6) | 61(24.4) | 0.956 |
|  |  |  | - | 174(60.21) | 90(31.14) | 25(8.65) |  | 438(75.78) | 140(24.22) |  |

Bold value means P < 0.05.
